# 

**Published:** 1877-06

**Authors:** 


### Meeting of the New York State Medical Society.

It is to be noticed that the Annual Meeting of the New York State Medical Society is to be held the third Tuesday of June—June 19th at 11 o'clock, A. M., in the city of Albany. It is desirable that all members and delegates should attend. We understand that the order of exercises will be determined previous to the meeting as far as it is possible to fore know what will be presented, and that thus the interest and advantage of the meeting will be greatly augmented.

### American Gynæcological Society.

The Second Annual Meeting of this Society will be held in Boston, May 30th. The President, Dr. FORDYCE BARKER of New York, will give the Annual Address.

———:o:———

AMERICAN MEDICAL COLLEGES.—A meeting of the Provisional Association of American Medical Colleges will be held at the Palmer House, Chicago, on Saturday, June 2d, 1877, at 10 o'clock, A. M. All colleges represented at the meeting of the Association held June, 1876, are invited to send delegates to the ensuing meeting, and all chartered medical colleges in the United States recognized as "regular" by the colleges already represented in this Association, are also invited to send delegates from their Faculties to the said meeting.

J. B. BIDDLE, M. D., President.

———:o:———

PHILADELPHIA, April 12, 1877.

The interval between the issue of the First and Second Parts of DR. DUHRING'S ATLAS OF SKIN DISEASES has been so much longer than was expected, that the Publishers deem it due to the subscribers to explain that the delay has been occasioned by unlooked-for difficulties attending the reproduction of the portraits.

The Publishers would assure the subscribers that the Parts will in future be issued as rapidly as consistent with the perfection of the work.

J. B. LIPPINCOTT & CO.

### Books and Pamphlets Received.

The Practitioner's Handbook of Treatment or the Principles of Therapeutics. By J. Milner Fothergill, M. D. Philadelphia: Henry C. Lea, 1877. Buffalo: T. H. Butler.

The Microscopist, a Manuel of Microscopy. By J. H. Wythe, A. M., M. D. Philadelphia: Lindsay & Blakiston, 1877. Buffalo: T. H. Butler.

Diseases of the Skin. By Louis A. Duhring, M. D. Philadelphia: J. B. Lippincott & Co., 1877 Buffalo: T. H. Butler.

Electro-Thermal Bath. By Justin Hayes, M. D. Chicago: Jansen McClurg & Co., 1877.

A Directory for the Dissection of the Human Body. By John Cleland, M. D., F. R. S. Philadelphia: Henry C. Lea, 1877. Buffalo: T. H. Butler.

Transactions of the American Medical Association, Prize Essay Supplement to Vol. 27, 1876, Excision of the Large Joints of the Extremities, By H. Culberteon, M. D.

Atlas of Skin Diseases By Louis H. Duhring, M. D. Part II. Philadelphia: J. H. Lippincott & Co., 1877.

A Course of Practical Histology. By E. A. Schafer. Philadelphia: Henry C. Lea, 1877. Buffalo: T. H. Butler.

Classification of Skin Diseases. By L. Duncan Buckley, A. M., M. D.

Two Cases of Morphœ. By I. Duncan Buckley, A. M., M. D.

### Contents of June Number.

### ORIGINAL.

THE BUFFALO

**Medical and Surgical Journal**

PUBLISHED MONTHLY,

**Containing Original Articles, Reports of Medical Societies and Hospitals, Editorial, Reviews, Correspondence, Army News, etc., etc.; including the usual variety of Medical Periodical Publications. Specimen copies sent on application.**

Address Buffalo Medical & Surgical Journal, Buffalo, N. Y.

TERMS OF SUBSCRIPTION, $3.00 PER YEAR, IN ADVANCE.

